# Rheological and Functional Properties of Dark Chocolate with Partial Substitution of Peanuts and Sacha Inchi

**DOI:** 10.3390/foods11081142

**Published:** 2022-04-15

**Authors:** Lucero Quispe-Chambilla, Augusto Pumacahua-Ramos, David Choque-Quispe, Francisco Curro-Pérez, Hilka Mariela Carrión-Sánchez, Diego E. Peralta-Guevara, Mery Luz Masco-Arriola, Henry Palomino-Rincón, Carlos A. Ligarda-Samanez

**Affiliations:** 1 Food Science and Technology, Universidad Nacional de San Antonio Abad del Cusco, Cusco 08000, Peru; lucerito8989@gmail.com; 2 Department of Food Engineering, Universidad Nacional Intercultural de Quillabamba, Cusco 08000, Peru; francisco.curro@uniq.edu.pe (F.C.-P.); hilka.carrion@uniq.edu.pe (H.M.C.-S.); 3 Agroindustrial Engineering, Universidad Nacional José María Arguedas, Andahuaylas 03701, Peru; dchoque@unajma.edu.pe (D.C.-Q.); hpalomino@unajma.edu.pe (H.P.-R.); caligarda@unajma.edu.pe (C.A.L.-S.); 4 Water Analysis and Control Research Laboratory, Universidad Nacional José María Arguedas, Andahuaylas 03701, Peru; diepltagvra@gmail.com; 5 Department of Chemical Engineering, Universidad Nacional de San Antonio Abad del Cusco, Cusco 08000, Peru; mery.masco@unsaac.edu.pe

**Keywords:** cocoa, Sacha Inchi, peanut, fatty acids, activation energy, rheological properties

## Abstract

Chocolate is a widely consumed product, due to the contribution of fats and antioxidant compounds; the addition of other components makes it possible to increase the content of polyunsaturated fatty acids, although they can affect its rheological properties. The influence of the partial addition of peanut paste and Sacha Inchi on the rheological and functional properties of dark chocolate was evaluated. Cocoa beans, peanuts, and Sacha Inchi were refined in order to obtain the cocoa paste (PC), peanut paste (PM), and Sacha Inchi paste (PSI). Then, mixtures between 0 to 20% of PM and PSI were formulated, and the rheological properties were evaluated at 30, 40, and 50 °C; these were adjusted to mathematical models. Functional groups were identified by FTIR in ATR mode, and it was observed that the partial addition of PM and PSI did not show significant changes in the shear stress and apparent viscosity of the mixture, although they did show dependence on temperature. The Herschel–Bulkley model showed a better adjustment (R^2^ > 0.999), reporting behavior index values, *n* < 1.0, and indicating pseudo-plastic behavior for pastes and formulations. The yield limit $\tau_{y}$ and the consistency index k_H_ increased significantly with the addition of PM and PSI, but they decreased with increasing temperature. The activation energy show values between 13.98 to 18.74 kJ/mol, and it increased significantly with the addition of PM and PSI. Infrared analysis evidenced the presence of polyunsaturated fatty acids, coming mainly from PSI and PM. The addition of PM and PSI does not influence the rheological properties and allows for an increase in the content of polyunsaturated fatty acids.

## 1. Introduction

The cocoa (*Teobroma cacao* L.) Chuncho is grown in the province of Quillabamba, Cusco, Peru. It is very representative because it is rich in total fatty acids and other bioactive compounds, unlike other hybrid varieties [1,2], due to its higher content of polyphenols and compounds antioxidants [3,4].

The chocolates are processed with cocoa beans, and their quality depends on their formulation; a content greater than 35% of cocoa liquor is qualified as dark chocolate [5,6]. This product is a source of carbohydrates and fats (saturated 59.7% and monounsaturated 38.7%) and, in greater quantity, oleic acid (38.6%) [5,7,8].

In the production of chocolates, many substitute and addition compounds are integrated in order to give it greater added value and special characteristics; generally being the addition of products with a high content of unsaturated fat such as almonds, peanuts, chestnuts, among others, allowing it to improve its nutritional contribution [9,10]. This addition of fatty material in dark chocolates can be up to 5%, to guarantee its authenticity, since higher percentages suggest adulterated chocolate; on the other hand, it is related to the stability of the fats during storage [8,11,12].

The Sacha Inchi (*Plukenetia volubilis* L.), is an endemic plant of the South American Amazon, mainly cultivated in the inter-Andean valleys in Peru [13,14,15,16]. It has an oilseed with a high content of bioactive compounds, tocopherols, and sterols [17,18], especially polyunsaturated fatty acids (91.6%), highlighting linolenic acid (48.2%), α-linoleic acid (34.1%), and oleic acid (8.9%), which are essential fatty acids for humans [19,20,21,22].

The peanut (*Arachis hypogaea* L.), whose fruit is an oilseed or legume, is mainly consumed as peanut butter [23] or as oil directly. It has a high content of bioactive compounds [24,25,26], including niacin, folic acid, vitamin E, phytosterols, and phenolic compounds, and about 56% oil, mainly oleic and α-linoleic acid, which represent about 75% of the oil [26,27,28,29,30].

The development of the formulation and substitution of the different chocolates and how this factor intervenes in the rheological characteristics that accompany their production are still a challenge [11,31]. As it presents a non-Newtonian behavior, chocolate is defined by the elastic limit and the plastic viscosity, which are related to the energy necessary to initiate and maintain the flow [31,32].

During chocolate processing, factors such as the amount of fat, the type of fat, particle size, and emulsifiers influence the behavior of the rheological properties profile [33,34,35], and it may affect the operability of the production stages, such as transportation, pumping, during mixing, or in industrial applications such as molding or coating [11,36,37].

The formulation of partially substituted chocolates is related to the size of the particles and the amount of fat, which are conditioning factors for the rheological characteristics (viscosity and elastic limit) [31,38], and capillary flow [39]. Thus, the addition of pastes with a high content of solids and polyunsaturated fatty acids such as Sacha Inchi, and peanuts, improves the chocolate texture [20,40].

The aim of this research was to elaborate on dark chocolate partially substituted with Sacha Inchi and peanut paste, study the rheological behavior and characterize the functional groups by infrared analysis, of raw materials and products.

## 2. Materials and Methods

### 2.1. Raw Material

Whole cocoa beans of the Chuncho variety were used. The grains were brought from the Convencion province in Cusco, Peru. It is located at 12°51′32″ S, 72°42′02″ W, and 1036 m of altitude. On the other hand, the peanut and Sacha Inchi grains were obtained from Yomentoni in Echarati, which is located at 12°34′52″ S, 73°00′39″ W, and 550 m of altitude, Cusco, Peru.

### 2.2. Preparation of Cocoa, Peanut, and Sacha Inchi Pastes

Cocoa beans, peanuts, and Sacha Inchi were roasted in an IMACO model HEB25R25L tray oven at 120 °C/15 min, 135 °C/30 min, and 135 °C/35 min, respectively. Then, they were manually shelled and pre-ground in a blender OSTER Brand. The samples were independently refined in a Premier-PG508 stone roller refining mill, and the samples were added to sugar at 30% (p/p). The cocoa paste (PC) was refined for 72 h, 48 h for peanut paste (PM) and Sacha Inchi paste (PSI), then it was sieved through a 450 ASTM mesh (<30 µm).

### 2.3. Black Chocolate Elaboration Process

The partially substituted dark chocolates were prepared using a mixed design (Table 1). The pastes were mixed in an isothermal bath at 50 °C with continuous stirring. Subsequently, they were immediately cooled up to 27 °C and then heated up to 30 °C in order to favor the crystallization process. After that, they were molded and taken to a cold chamber at 8 °C for 6 h, and then stored at 11 °C for subsequent analysis.

### 2.4. Analysis of Rheological Properties

The experimental samples were subjected to continuous tests through an Anton Paar rotational rheometer, model MCR702e, using concentric cylinder geometry at a controlled shear rate of 1 to 50 s^−1^ and 30, 40, and 50 °C [41]. The data were analyzed using the shear stress models for non-Newtonian fluids through the Power-Law, Bingham Plastic, Herschel–Bulkley, and Casson [32,33,35,36,37], and these models are shown in Table 2.

### 2.5. Modeling of Rheological Models

The rheological models were adjusted using the Statistica 8.0 software (Statsoft, Tulsa, OK, USA). The convergence criteria used were the adjusted correlation coefficient (R^2^), the residual mean square of the error—*RMSE* (Equation (5)), and the mean absolute percent of the error—*MAPE* (Equation (6))

(5) $$RMSE=\frac{\sum_{i}{\left(x_{i}-{\hat{x}}_{i}\right)}^{2}}{n}$$

(6) $$MAPE=\frac{100}{N}\overset{N}{\underset{i=1}{\sum }}\frac{\left|x_{i}-{\hat{x}}_{i}\right|}{x_{i}}$$

where: $x_{i}$, observed value; ${\hat{x}}_{i}$, predicted value; n, number of observations; N, number of experimental observations.

### 2.6. Determination of Activation Energy

The activation energy is the energy necessary to allow the flow of the pastes, and it describes the thermodynamic behavior of the apparent viscosity, that is, when the viscous forces are overcome by the kinetic energy due to the efforts and movements, giving rise to the decrease in the resistance to flow [42].

Thus, the effect of temperature on the rheological behavior, was evaluated through the activation energy ($E_{a}$), and it was calculated using the Arrhenius equation (Equation (7)), from the apparent viscosity.

(7) $$ln\mu =ln\mu_{0}-\frac{Ea}{R}.\frac{1}{T}$$

where: μ, is the viscosity; $\mu_{0}$, pre-exponential factor; *R*, universal gas constant (8.314 kJ/kmol K); *T*, absolute temperature, K.

### 2.7. Infrared Analysis of Dark Chocolate

The chocolate pastes and samples were analyzed in a Fourier transform infrared (FTIR) spectrometer (Thermo Scientific Nicolet iS50 model, Waltham, MA, USA). The analysis was carried out in ATR mode controlled by the OPNIC software in the range of 4000 to 400 cm^−1^. The analyzed data represented the average of three repetitions to each experimental sample, which was collected at a resolution of 4 cm^−1^.

## 3. Results and Discussion

### 3.1. Rheological Characterization

**Figure 1:**
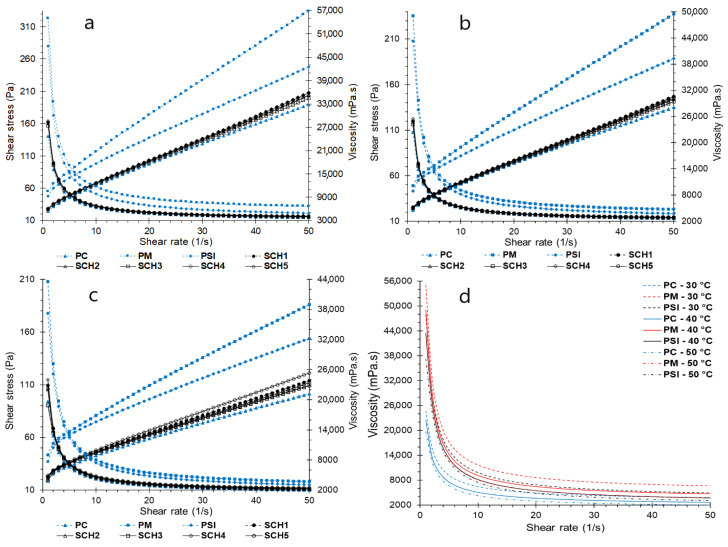
Curves of apparent viscosity and shear stress t for formulations (**a**) 30 °C, (**b**) 40 °C, (**c**) 50 °C, (**d**) apparent viscosity of paste and formulations.

The experimental behavior of the shear stress and apparent viscosity in the dark chocolate pastes and formulations at temperatures of 30, 40, and 50 °C are shown in Figure 1.

It was observed that the shear stress decreases rapidly with the increase in the shear rate for pastes and formulations at the study temperatures. However, PC and the different formulations show a more pronounced decrease in comparison with PSI and PM (Figure 1). On the other hand, viscosity increases considerably with the shear rate, but for PC and formulations, the viscosity values are lower compared to PSI and PM.

The fact that the shear stress and viscosity curves are similar for PC and the formulations are due to the behavior of chocolate which presents thinning due to shear stress [31,43]. This can be seen in Figure 1a–c, where it is evident that the shear stress is lower. On the other hand, the effect of temperature on the shear stress is inversely proportional, while the viscosity reported lower values with the increase in temperature (Figure 1d). This showed non-ideal elastic behavior, which is characteristic of chocolate paste, and other pastes with a high-fat content [25,33,34].

### 3.2. Rheological Modeling and Incidence of Temperature

It was observed that the models described in Table 2 reported R^2^ values > 0.99; however, the Herschel–Bulkley model reported lower MAPE values < 1.27 and RSME < 2.10 (Table 3), and this model is widely used in the study of the behavior of chocolate paste with partial substitutions [35,44,45,46].

**Figure 2:**
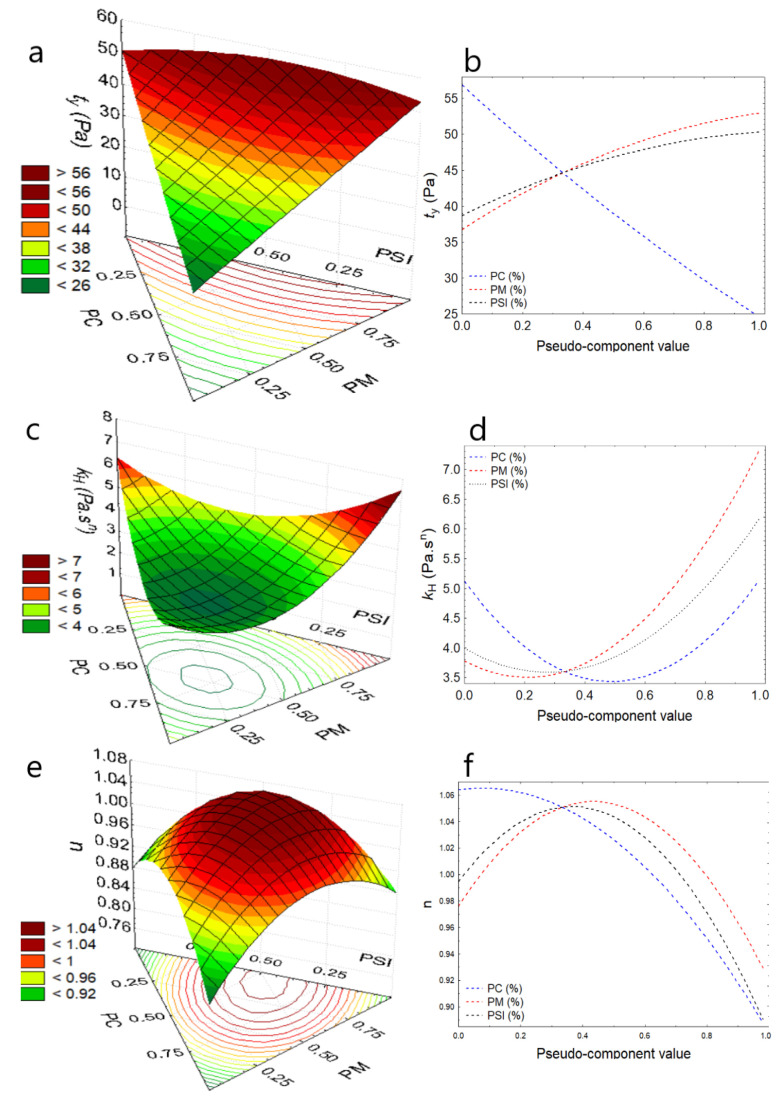
Response surface and component effect at 30 °C (**a**) $\tau_{y}$, (**b**) effects for $\tau_{y}$, (**c**) k_H_, (**d**) effects for k_H_, (**e**) *n*, (**f**) effects for *n*.

**Figure 3:**
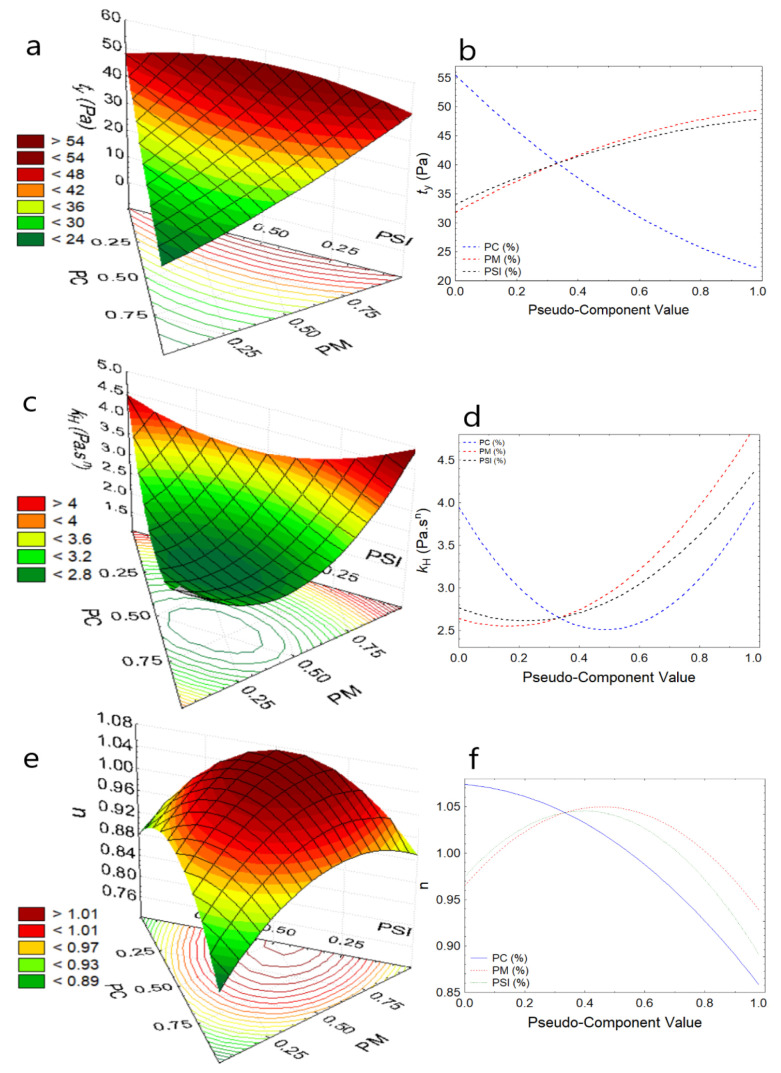
Response surface and component effect at 40 °C (**a**) $\tau_{y}$, (**b**) effects for $\tau_{y}$, (**c**) k_H_, (**d**) effects for k_H_, (**e**) *n*, (**f**) effects for *n*.

**Figure 4:**
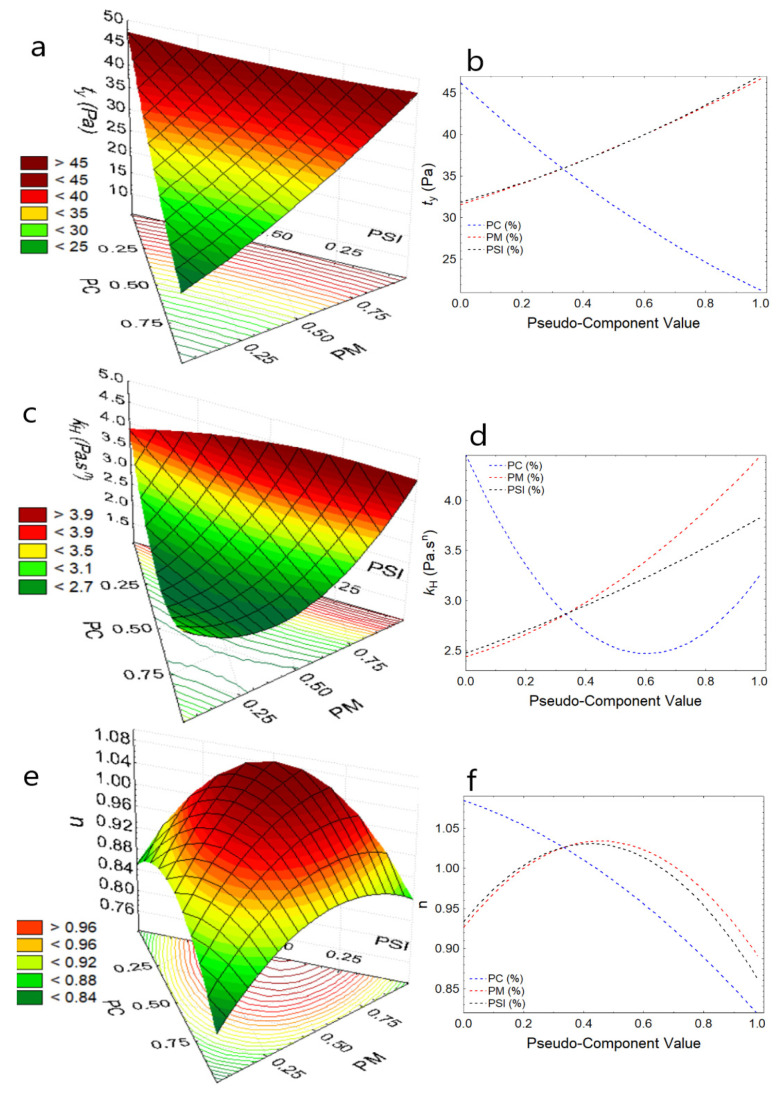
Response surface and component effect at 50 °C (**a**) $\tau_{y}$, (**b**) effects for $\tau_{y}$, (**c**) k_H_, (**d**) effects for k_H_, (**e**) *n*, (**f**) effects for *n*.

Regarding the elastic or yield stress, $\tau_{y}$ reported values between 24.56 to 32.08 Pa for the formulations, and SCH4 (75% PC, 12% PM, and 14% PSI) presented higher values (Table 3). It was observed that the increase in PM increases the shear stress for the study temperatures while any increase in PSI makes $\tau_{y}$ high (Figure 2a, Figure 3a and Figure 4a); it is because PSI and PM provide a large number of solids to chocolate, due to the higher dry matter content compared to cocoa [47,48]. In contrast, the increase in PC reduces the yield stress for the study temperatures (Figure 2b, Figure 3b and Figure 4b), due to the high content of unsaturated fats that act as dispersants. So, the analysis of this parameter provides information on the maneuverability during chocolate processing, especially when substituents with high dry matter content are added [11,32,33,47,49], this behavior was observed by Medina-Mendoza et al., using Sacha Inchi oil [40].

Regarding the consistency index, k_H_, which is an indirect measure of viscosity, it was observed that values are between 2.43 and 4.21 Pa·s^n^ for the formulations (Table 3), and the increase in PSI and PM considerably increase the consistency of the chocolate at the study temperatures (Figure 2c, Figure 3c and Figure 4c), due to the addition of solids in the dark chocolate matrix. This limits the mobility of the particles [37,43,49], which is normal behavior for food products that are not chemically pure or physically homogeneous [47,48,50,51], while the addition of PC reports a slight increase in k_H_ (Figure 2d, Figure 3d and Figure 4d).

Behavior index values, *n* < 1.0 are considered fluids with pseudoplastic behavior, and it was observed that as PC, PSI, and PM increase, dark chocolate pastes show greater pseudoplasticity, indicating that they move away from more than 1.0, and this behavior is more attenuated with the increase in temperature (Figure 2e, Figure 3e and Figure 4e), increasing its apparent viscosity considerably. This behavior is not desirable during the processing and packaging of dark chocolate paste due to the difficulty of maneuverability [11,52].

The independent analysis for PC reported that the Casson model adjusts better to the experimental data with R^2^ > 0.99, RSME < 0.84, and MAPE < 0.99 (Table 4). It is also observed that k, consistency index, decreases with the increase in temperature. PC shows considerable deformation, increasing its creep, while $\tau_{y}$ increases significantly; that is, a greater effort is required to deform the PC with the increase in temperature. However, this value is always lower than the reported by the Bingham model (Table 4). This is because the melting point of the fatty acids present, which cause a decrease in the particle size in the mass of PC [33,34,35,36,38].

### 3.3. Activation Energy

The resistance to flow was evaluated through the activation energy [42]. It was observed that PM and PSI reported higher values of E_a_ 21.04 and 20.19 kJ/mol, respectively (Table 3); this is because these pastes have a higher content of fatty acids compared to PC, which is associated with fatty acids with less unsaturation, giving it less viscosity and allowing to reduce creep. Accordingly, high values of E_a_ are related to the change in the viscosity of products due to the increase in temperature variation [43,53].

**Figure 5:**
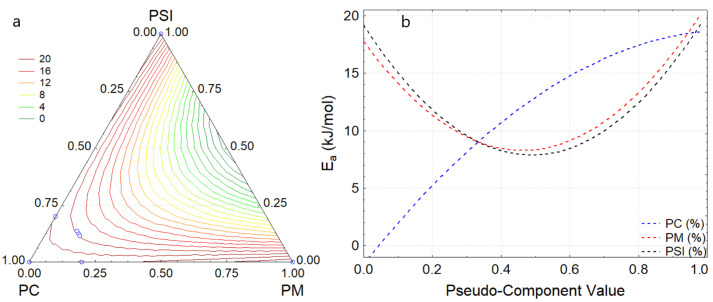
(**a**) response Surface for Ea, (**b**) effect of the components for E_a_.

On the other hand, the formulations reported E_a_ values between 13.98 to 18.74 kJ/mol, observing that the addition of PM and PSI considerably increased E_a_ at a similar intensity (Figure 5a), presenting lower E_a_ for proportions between 40 to 60% (Figure 5b), which suggests less energy application during processing while the effect of PC addition increases E_a_ in dark chocolate.

### 3.4. IR Analysis

**Figure 6:**
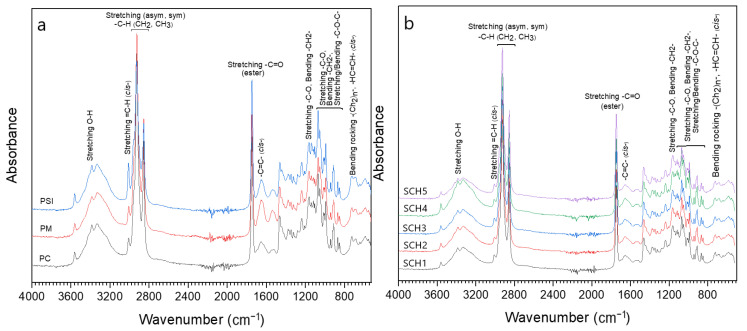
Vibrational modes (**a**) PC, PM y PSI, (**b**) SCH1, SCH2, SCH3, SCH4, SCH5.

Chocolate is a product rich in sugars, and it presents characteristic peaks of carbohydrate molecules, located between 1150–825 cm^−1^, which are related to stretching vibrations of the C-O group, rolling vibrations of the CH group, and stretching and rolling vibrations of the C-O-C bond (Figure 6). In the same way, spectra were observed around 3330 cm^−1^, corresponding to the asymmetric stretching vibration of the C-H bond [54,55]. These regions show the highest intensity peaks corresponding to PSI, as well as formulations SCH4 and SCH3 that contain a higher percentage of PSI, which suggests that Sacha Inchi grains provide a higher sucrose content [14,15,18,19].

Around 720 cm^−1^, a band is observed, although more pronounced for PSI; this results from the oscillating vibration of methylene and the out-of-plane bending of cis-disubstituted olefins [55,56], and these decrease considerably for the formulations; this would be due to the higher percentage of PC aggregates and the chocolate transformation process; likewise, the peaks around 1370 cm would be related to the symmetric bending vibration of the methyl groups [55], whose intensity decreases for the formulations.

On the other hand, the region associated with the peak 2924 and 2853 cm^−1^ are related to the functional groups -CH_2_ in a vibrational mode of symmetric and asymmetric stretching [54]; these would be linked to carbohydrates and lipids in dark chocolate [7,8].

In addition, three regions were observed at 1655, 1750–1730, 3010 cm^−1^, which are linked functional groups -C=O (ester and acid), -C=C-(form cis), and =C–H (form cis), which correspond to lipids present in pastes and formulations (Table 5), which would allow determining the fat quality due to the triglyceride content, the form of unsaturation of acyl groups, and the chain length of fatty acids in their constitution [55,57,58].

A band was observed around 3010 cm^−1^ (Figure 6), which is associated with the stretching vibration of cis olefinic double bonds (=C–H) of α-3 polyunsaturated fatty acids (PUFA) [54,58,59], with greater intensity for PSI, and this is due to the content of polyunsaturated acids such as linolenic acid (α-3) and linoleic acid (α-6), present in the Sacha Inchi seed [14,19,20]. This behavior influences the SCH4 and SCH3 samples, while PC and PM show less intensity; this is due to the lower content of linoleic acid (α-6) in cocoa and peanut seeds [23,24,25,26,29,30], which influences the SCH1, SCH2, and SCH5 samples.

Regarding the bands around 2900 cm^−1^ corresponding to the asymmetric and symmetric -CH stretching of sugars such as sucrose [60], and long-chain saturated fatty acids mainly from cocoa and peanuts [7,30,55,56], It was observed that the peaks are similar and with high intensity, in the order PSI > PM > PC (Table 5), while in the prepared samples the intensity decreases in a similar magnitude (Figure 6).

The band corresponding to the ester carboxyl group, C=O of triacylglycerides, is shown in the region between 1767–1716 cm^−1^, and it allows discrimination of the samples based on their lipid matrix [56,59]. Furthermore, it is related to the formation of free fatty acids [61,62], and it was observed at high intensity in the order PM > PSI > PC, which shows a high content of fatty acids susceptible to oxidation [8,61]. About the formulations, it was observed that they present similar intensity, although lower than pure pastes. This decrease is due to the processing of dark chocolate, where fatty acids are modified due to temperature and atmospheric oxygen, which decrease their unsaturations [3,19,30,54,55,59,61].

The region that represents the Cis double bonds associated with the stretching vibration of the C=C bond is around 1650 cm^−1^. It showed greater intensity for PM > PSI > PC (Table 5), while the prepared samples showed dependence with the addition of PSI and PM.

## 4. Conclusions

The partial substitution of dark chocolate paste (PC) for peanut paste (PM) and Sacha Inchi paste (PSI) did not show significant changes in shear stress and apparent viscosity of the mixture, although these show dependence on temperature. The yield stress modeling (τ), and the shear rate (γ), were adequately adjusted to the Herschel–Bulkley model, which reported behavior index values, *n* < 1.0, and it shows a pseudo-plastic behavior for the pastes and formulations. The yield limit, $\tau_{y}$, and the consistency index, k_H_, increased significantly with the addition of PM and PSI, while they decreased with increasing temperature. The activation energy increases significantly with the addition of PM and PSI. Infrared analysis evidenced the increase in polyunsaturated fatty acids in dark chocolate, mainly from PSI and PM.

## Figures and Tables

**Table 1:** Partially substituted dark chocolate formulation.

Formulation	PC (%)	PM (%)	PSI (%)
PC	100.0	0.0	0.0
PM	0.0	100.0	0.0
PSI	0.0	0.0	100.0
SCH1	75.0	12.5	12.5
SCH2	80.0	20.0	0.0
SCH3	80.0	0.0	20.0
SCH4	75.0	11.5	13.5
SCH5	75.0	13.5	11.5

Where: PC, cocoa paste; PM, peanut paste; PMI, Sacha Inchi paste; SCH, substituted dark chocolate.

**Table 2:** Rheological models for non-Newtonian fluids.

Model	Equation	Parameters	
Power-Law	$\tau =k\gamma^{n}$	k, n	(1)
Bingham plastic	$\tau =\tau_{y}+\eta_{B}\gamma$	$\tau_{y},\;\eta_{B}$	(2)
Herschel–Bulkley	$\tau =\tau_{y}+k_{H}\gamma^{n}$	$\tau_{y},\;k_{H},\;n$	(3)
Casson	$\tau^{1/2}=\tau_{y}{}^{1/2}+{\left(k\gamma \right)}^{1/2}$	$\tau_{y},\;k$	(4)

where: τ, yield stress (Pa); γ, shear rate, (s^−1^); k, consistency index (Pa·s^n^); n, behavior index; $\tau_{y}$, lastic limit or yield point (Pa); $\eta_{B}$ plastic viscosity (Pa·s); $k_{H}$, consistency index (Pa·s^n^).

**Table 3:** Rheological parameters of the Herschel–Bulkley model, and activation energy.

	T (°C)	$\tau_{y}$	$k_{H}$	n	R^2^	RSME	MAPE	E_a_ (kJ/mol)
PC	30	24.15	5.28	0.88	0.9998	0.75	0.69	18.68
	40	22.00	4.12	0.85	0.9997	0.58	0.62	
	50	20.96	3.34	0.81	0.9992	0.69	0.70	
PM	30	53.04	7.52	0.92	0.9999	0.91	0.38	21.04
	40	49.49	4.99	0.93	0.9998	0.80	0.36	
	50	47.07	4.50	0.88	0.9992	1.15	0.51	
PSI	30	50.35	6.32	0.88	0.9995	1.36	0.67	20.19
	40	47.94	4.45	0.88	0.9990	1.30	0.56	
	50	47.51	3.86	0.85	0.9959	2.10	1.06	
SCH1	30	31.35	3.87	0.97	0.9994	1.37	1.33	13.98
	40	27.44	2.83	0.95	0.9993	0.98	1.14	
	50	24.98	2.75	0.89	0.9992	0.78	0.90	
SCH2	30	30.09	4.17	0.95	0.9995	1.23	1.19	18.67
	40	25.86	3.15	0.92	0.9995	0.83	0.96	
	50	24.81	2.64	0.88	0.9989	0.86	0.99	
SCH3	30	28.98	4.21	0.94	0.9995	1.13	1.12	17.93
	40	25.16	3.13	0.92	0.9996	0.74	0.89	
	50	24.56	2.71	0.88	0.9977	1.27	1.41	
SCH4	30	32.08	3.78	0.98	0.9994	1.38	1.27	17.98
	40	26.66	3.01	0.94	0.9995	0.83	0.91	
	50	26.04	2.43	0.91	0.9991	0.77	0.94	
SCH5	30	30.21	4.10	0.95	0.9995	1.26	1.19	18.74
	40	26.75	2.96	0.94	0.9994	0.88	1.04	
	50	26.01	2.59	0.92	0.9988	1.03	1.17	

**Table 4:** Parameters of the rheological models for PC.

**Power Law**					
**T (°C)**	k	n	**R^2^**	**RSME**	**MAPE**
30	13.91	0.66	0.995	3.56	4.33
40	13.16	0.59	0.992	2.92	4.30
50	13.15	0.51	0.990	2.36	4.09
**Bingham Equation**					
**T (°C)**	$\tau_{y}$	$\eta_{B}$	**R^2^**	**RSME**	**MAPE**
30	33.88	3.15	0.998	2.64	3.08
40	28.48	2.18	0.996	1.96	2.80
50	26.61	1.54	0.993	1.82	3.00
**Casson Equation**					
**T (°C)**	k	$\tau_{y}$	**R^2^**	**RSME**	**MAPE**
30	2.15	11.29	0.999	0.84	0.99
40	1.29	12.63	0.999	0.53	0.74
50	0.81	13.60	0.999	0.43	0.76

**Table 5:** Vibrational modes for dark chocolate pastes and formulations.

Samples	Peak (cm^−1^)	Vibrational Mode	Main Attribution	Region (cm^−1^)	Area
PC	3005.22	=C–H	PUFA α-3	3030–2990	0.37
PM	3007.83	=C–H	PUFA α-3	3030–2990	0.40
PSI	3010.46	=C–H	PUFA α-3	3030–2990	0.88
SCH1	3008.80	=C–H	PUFA α-3	3030–2990	0.23
SCH2	3006.82	=C–H	PUFA α-3	3030–2990	0.19
SCH3	3007.91	=C–H	PUFA α-3	3030–2990	0.25
SCH4	3009.24	=C–H	PUFA α-3	3030–2990	0.21
SCH5	3007.30	=C–H	PUFA α-3	3030–2990	0.24
PC	1746.63	C=O	Triacylglycerides	1767–1716	4.02
PM	1746.68	C=O	Triacylglycerides	1767–1716	3.96
PSI	1746.18	C=O	Triacylglycerides	1767–1716	4.06
SCH1	1746.71	C=O	Triacylglycerides	1767–1716	4.02
SCH2	1746.73	C=O	Triacylglycerides	1767–1716	3.96
SCH3	1746.70	C=O	Triacylglycerides	1767–1716	3.98
SCH4	1746.70	C=O	Triacylglycerides	1767–1716	4.04
SCH5	1746.67	C=O	Triacylglycerides	1767–1716	4.00
PC	1656.74	C=C	Cis double bonds	1712–1585	1.24
PM	1651.76	C=C	Cis double bonds	1712–1585	2.68
PSI	1651.79	C=C	Cis double bonds	1712–1585	1.82
SCH1	1656.18	C=C	Cis double bonds	1712–1585	1.08
SCH2	1655.77	C=C	Cis double bonds	1712–1585	1.19
SCH3	1655.74	C=C	Cis double bonds	1712–1585	1.16
SCH4	1653.35	C=C	Cis double bonds	1712–1585	1.53
SCH5	1652.74	C=C	Cis double bonds	1712–1585	1.11

## Data Availability

The data presented in this study are available in this same article.
